# Automatic Measurement of Chew Count and Chewing Rate during Food Intake

**DOI:** 10.3390/electronics5040062

**Published:** 2016-09-23

**Authors:** Muhammad Farooq, Edward Sazonov

**Affiliations:** Department of Electrical Engineering, The University of Alabama, Tuscaloosa, AL 35487, USA

**Keywords:** chewing rate, food intake detection, piezoelectric sensor, artificial neural network, feature computation, chew counting, peak detection

## Abstract

Research suggests that there might be a relationship between chew count as well as chewing rate and energy intake. Chewing has been used in wearable sensor systems for the automatic detection of food intake, but little work has been reported on the automatic measurement of chew count or chewing rate. This work presents a method for the automatic quantification of chewing episodes captured by a piezoelectric sensor system. The proposed method was tested on 120 meals from 30 participants using two approaches. In a semi-automatic approach, histogram-based peak detection was used to count the number of chews in manually annotated chewing segments, resulting in a mean absolute error of 10.40% ± 7.03%. In a fully automatic approach, automatic food intake recognition preceded the application of the chew counting algorithm. The sensor signal was divided into 5-s non-overlapping epochs. Leave-one-out cross-validation was used to train a artificial neural network (ANN) to classify epochs as “food intake” or “no intake” with an average *F*1 score of 91.09%. Chews were counted in epochs classified as food intake with a mean absolute error of 15.01% ± 11.06%. The proposed methods were compared with manual chew counts using an analysis of variance (ANOVA), which showed no statistically significant difference between the two methods. Results suggest that the proposed method can provide objective and automatic quantification of eating behavior in terms of chew counts and chewing rates.

## 1. Introduction

Excessive eating can cause a drastic increase in weight; therefore, it is important to study the eating behavior of individuals to understand eating patterns contributing towards obesity. Similarly, people suffering from eating disorders experience changes in their normal eating patterns, causing individuals to either eat excessive or insufficient food compared to their body energy requirements. Obesity and eating disorders present a significant public health problem. Statistics from the World Health Organization (WHO) shows that worldwide, obesity is the fifth largest cause of preventable deaths [1]. People with anorexia nervosa have a shorter life expectancy (about 18 times less) in comparison to the people who are not suffering from this condition [2]. People suffering from binge-eating are at higher risk of cardiovascular diseases and high blood pressure [2]. Therefore, there is a need to study the food intake patterns and eating behavior of individuals to better understand patterns and factors contributing to the obesity and eating disorders.

Traditional methods for monitoring food intake patterns such as food frequency questionnaires, food records, and 24-h food recall rely on self-report of the participants [3–5]. Research suggests that, during self-report, participants tend to underestimate their intake where the underestimation varies between 10% and 50% of the food consumed [6]. Apart from being inaccurate, self-reporting also puts the burden on the patients because of the need for their active involvement [6]. Therefore, recent research efforts have focused on the development of methods and techniques that are accurate, objective, and automatic and reduce the patient’s burden of self-reporting their intake.

A number of wearable sensor systems and related pattern recognition algorithms have been proposed for monitoring ingestive behavior. The systems relying on chewing as an indicator of food intake use such sensor modalities as in-ear miniature microphones [7,8], accelerometers [9], surveillance video cameras [10], and piezoelectric sensors on the throat [11] or on the jaw [12–14]. In [7], chewing sounds recorded with a miniature microphone in the outer-ear were used to train hidden Markov models (HMMs) to differentiate sequences of food intake from sequences of no intake with an accuracy of 83%. In [15], an earpiece consisting of a 3D gyroscope and three proximity sensors that detected chewing by measuring ear canal deformations during food intake was proposed. Participant-dependent HMMs achieved an average classification accuracy of 93% for food intake detection. In [9], the use of a single axis accelerometer placed on the temporalis was proposed to monitor chewing during eating episodes in laboratory experiments. Several different classification techniques (decision tree (DT), nearest neighbor (NN), multi-layer perceptron (MLP), support vector machine (SVM) and weighted SVM (WSVM)) were compared, and WSVM achieved the highest accuracy of about 96% for detection of food intake. Our research group has been developing systems for monitoring ingestive behavior via chewing [12–14,16]. In [12], features computed from piezoelectric film sensors placed below the ear were used to train SVM and artificial neural network (ANN) models to differentiate between epochs of food intake and no intake with average accuracies of 81% and 86%, respectively. In [13], this system was tested by 12 participants in free living conditions for 24 h each. Sensor signals were divided into 30-s epochs, and for each epoch a feature vector consisting of 68 features was computed. Participant-independent ANN models differentiated between epochs of food intake and no intake with an average accuracy of 89% using leave-one-out cross-validation.

In recent years, researchers have focused on the automatic detection of chewing, but little work has been done on the quantification of chewing behavior, which may be an important factor in studying energy intake. Although no direct relationship has been established between obesity and chewing patterns, several studies have shown that increased mastication before swallowing of the food may reduce the total energy intake [17–21]. Results in [18] showed that obese participants had higher intake rates with lower numbers of chews per 1 g of food (pork pie) compared with the normal weight group. They also showed that an increase in the number of chews per bite decreased final food intake in both obese and normal weight participants. In [19], 45 participants were asked to eat pizza over four lunch sessions. Participants were asked to have 100%, 150%, and 200% of the number of chews of their baseline number (first visit) of chews per bite before swallowing. According to the authors, food intake (total mass intake) reduction of 9.5% and 14.8% was observed for chewing rates of 150% and 200%, respectively, compared with the 100% session. Our research demonstrated that the number of chews per meal may be used in estimating the energy intake if the energy density of the food is given. In [22], individually calibrated models were presented to estimate the energy intake from the counts of chews and swallows.

Most of these studies relied on the manual counting of chews either by the participants or by the investigators, either from videotapes or by watching participants in real time. Thus, there is a need to develop methods to provide objective and automatic estimation of chew counts and chewing rates. In recent studies, semi-automatic chew counting systems utilizing piezoelectric strain sensors have been proposed [23–25]. In [23,24], a modified form of the sensor system proposed in [13] was used to quantify the sucking count of 10 infants by using zero crossing. In [25], an algorithm was proposed for counting chews from a piezoelectric strain sensor and printed sensor signals. A group of five adult participants counted the number of chews taken while eating three different food items and marked each chewing episode with push button signals. A peak detection algorithm was used to count chews from known chewing segments (based on push button signals) with a mean absolute error of 8% (for both sensors). An example of chewing sound use, the chewing rate and bite weight for three different food types was estimated from the sounds captured by a miniature microphone in [8]. A possible limitation of the acoustic-based approach is its sensitivity to the environmental noise, which might require a reference microphone for noise cancellation [7]. For these systems to be useful in free living conditions, fully automated solutions are needed which can not only automatically recognize chewing sequences but can also quantify chewing behavior in terms of chew counts and chewing rates.

The goal of this paper is to present a method for automatic detection and quantification of the chew counts and chewing rates from piezoelectric film sensor signals. Main contributions of this work include the design of the proposed system as means of automatic and objective quantification of chewing behavior (chew counts and chewing rates), which could be used in studying and understanding the chewing behavior of individuals and its relation to the energy intake without relying on manual chew counts. Our previous research has shown that the piezoelectric strain sensor can be used for objective monitoring of eating in unrestricted free living conditions [13]. The approach presented in this work could be extended [21–26] to free living environments, studying the relation of chewing patterns, energy intake, and obesity in community-dwelling individuals. The system implements a fully automatic approach that first detects the intake of foods requiring chewing and then characterizes the chewing in terms of chew counts and chewing rates. Another contribution of this work is the testing of the piezoelectric sensor and related signal processing and pattern recognition algorithms in a relatively larger population in multi-day experiments with a wide variety of foods, which are representative of the daily diet.

## 2. Methods

### 2.1. Data Collection Protocol

For this study, 30 participants were recruited. The population consisted of 15 male and 15 female participants with an average age of 29.03 ± 12.20 years and a range of 19–58 years. The average body mass index (BMI) of the population (in kg/m^2^) was 27.87 ± 5.51 with a range of 20.5 to 41.7. Each participant came for four visits (a total of 120 experiments). Data from 16 experiments was discarded because of equipment failure. The remaining dataset consisted of a total of 104 visits/experiments. Recruited participants did not show any medical conditions which would hinder their normal eating or chewing. An Institutional Review Board approval for this study was obtained from Clarkson University, Potsdam, NY, and all participants signed a consent form before participation.

Participants were divided into three groups based on meal type, i.e., breakfast, lunch, and dinner, and were asked to make two different meal selections from the food items available at one of the cafeterias at Clarkson University to ensure that there was intra-subject variability in food selection. Overall, 110 distinct food items were selected by the participants, on average each participant consumed 1 to 3 food types and 1 or 2 different beverages. Representative food groups selected by the participants can be found in [22]. The wide spectrum of included food items ensures that the proposed algorithms behave well in the foods with varying physical properties eaten by the general population in their daily routine.

The meals were consumed during a visit to a laboratory instrumented for the monitoring of food intake. An accurate and objective reference (gold standard) was needed for the quantification of chewing sequences. At present, obtaining an accurate reference in free living conditions is virtually impossible. Therefore, the study was conducted in a laboratory environment where close observation of the ingestion process was performed with the sensors and a video recording. During each visit, participants were initially instrumented with the sensors [27]. As the first step of the protocol, the participants were asked to remain in a relaxed seated position for 5 min. Second, they were given unlimited time to eat self-selected foods. Participants were allowed to talk, pause food intake, and move around (within the limitations imposed by the sensor system) during the experiment to replicate normal eating behavior. As the final step of the protocol, participants were asked to remain in a relaxed seated position for another 5 min.

### 2.2. Sensor System and Annotation

**Figure 1:**
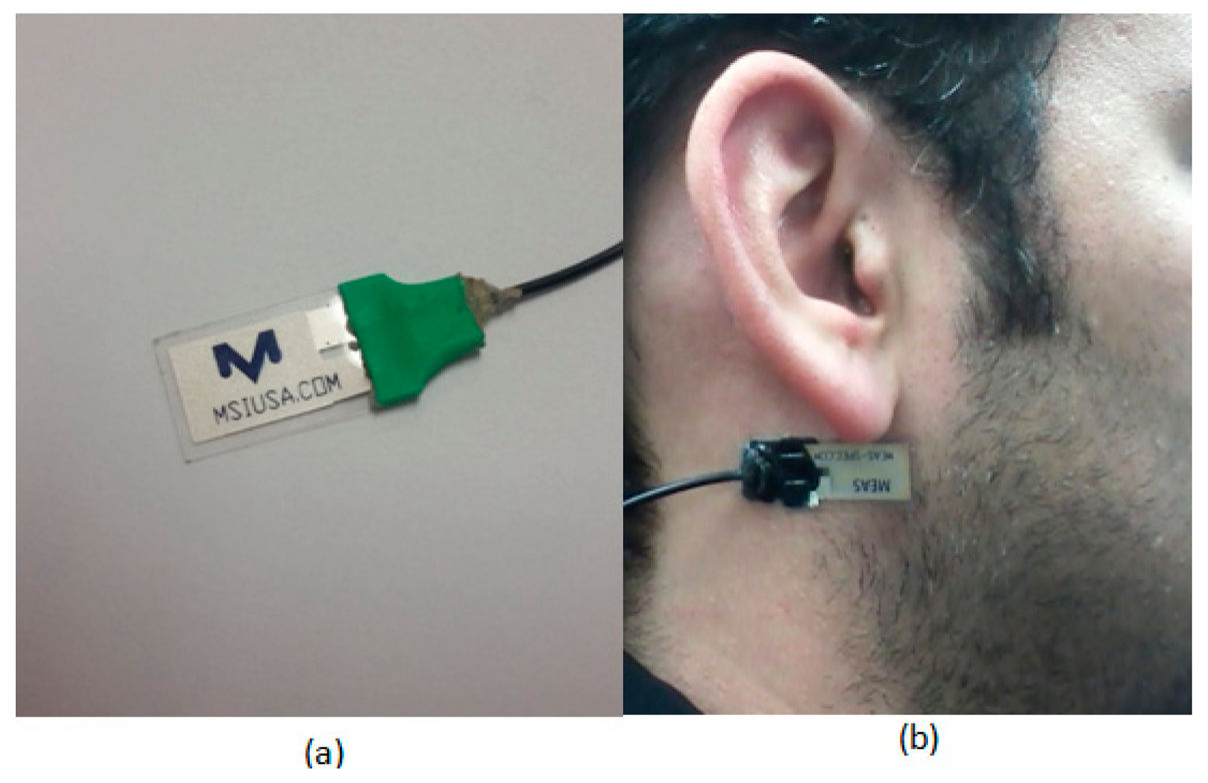
(**a**) Piezoelectric film sensor used in the study; (**b**) Sensor attached to a participant.

**Figure 2:**
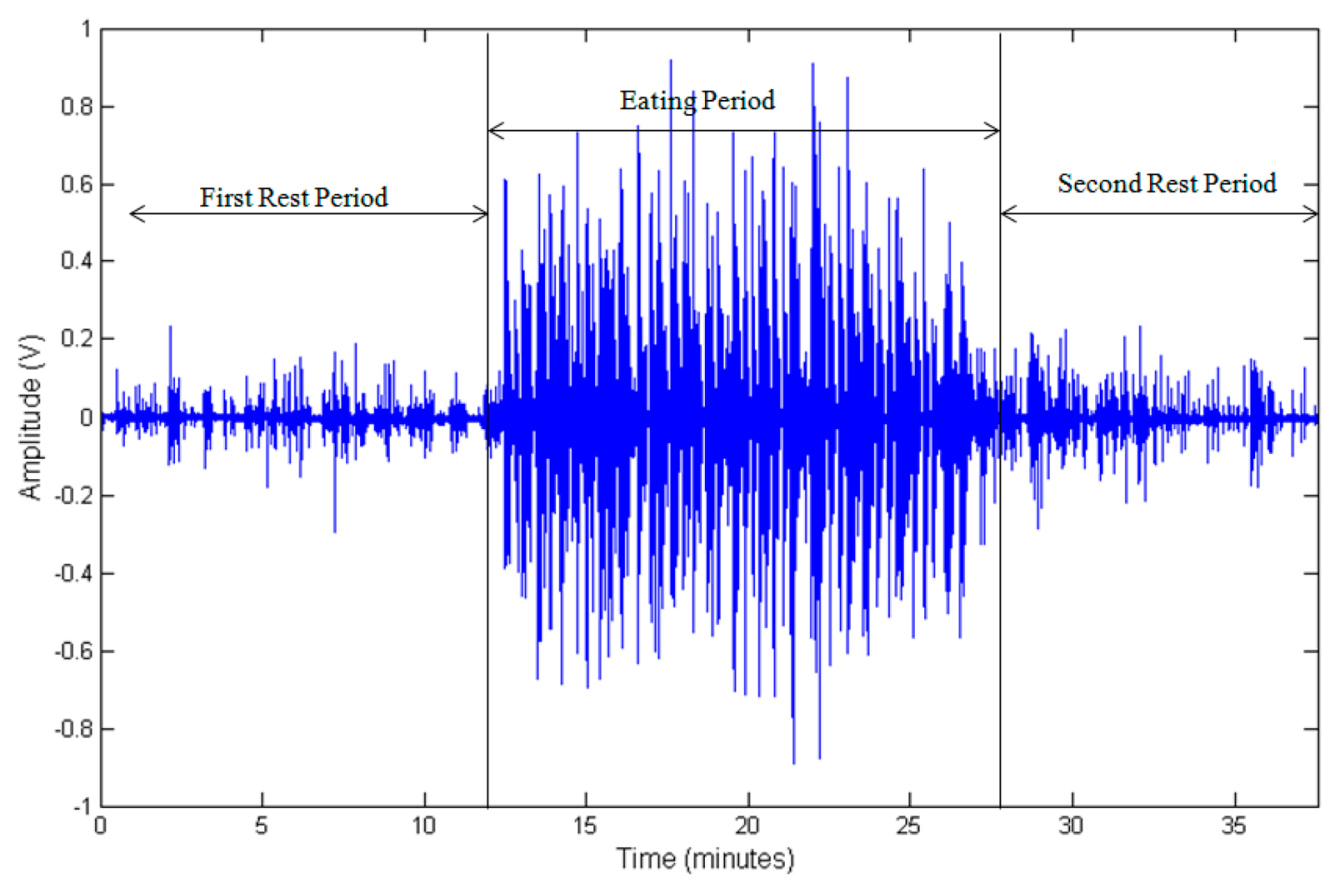
Piezoelectric sensor (raw) signal shows three parts of the experiment. First is a rest period, followed by an eating episode which is followed by a second rest period. Sampling frequency used was 44,100 Hz.

A multimodal sensor system was used to monitor participants [26]. A commercially available piezoelectric film sensor (LDT0-028K, from Measurement Specialties Inc., Hampton, VA, USA) was placed below the ear using a medical adhesive for capturing motions of the lower jaw during chewing/mastication of the food. The selected sensor is comprised of a piezoelectric PVDF polymer film (28-μm thickness) and screen-printed Ag-ink electrodes encapsulated in a polyester substrate (0.125-mm thickness). Vibration of the surface to which the sensor is attached creates strain within the piezo-polymer which in turn generates voltage. The selected sensor has a sensitivity of 10 mV per micro-strains, which has been shown to be enough to detect vibrations at the skin surface caused by chewing [26]. A custom-designed amplifier with an input impedance of about 10 MΩ was used to buffer sensor signals. Sensor signals were in the range of 0–2 V, were sampled at *f_s_* = 44,100 Hz with a data acquisition device (USB-160HS-2AO from the Measurement Computing) with a resolution of 16 bits, and were stored in computer memory. This sampling frequency ensures that the sensor will be able to pick speech signals. The total duration of the sensor signal data was around 60 h, and about 26 h of data belonged to food intake. Figure 1a, b shows an example of the piezoelectric film sensor and its attachment to a participant. Figure 2 shows an example of the sensor signal captured during the experiment.

Experiments were videotaped using a PS3Eye camera (Sony Corp., New York, NY, USA), and videos were time-synchronized with the sensor signals. Custom-built LabVIEW software was used to annotate videos and sensor signals [27]. During the annotation process, videos were reviewed by trained human raters. The custom-built software has the ability to play videos at different speeds and provided a timeline where human raters could mark the start and end of each eating event (bite, chewing, and swallows). The annotation software also allows the user to play the video frame by frame or any specific intervals. This enables the rater to watch the marked chewing segments multiple times for counting chews. Annotated start and end timestamps of chewing along with the corresponding chew counts were stored and used for algorithm development. Further details of the annotation procedure are described in [27]. Inter-rater reliability of the annotation procedure adopted here was established in a previous study, where three raters achieved an intra-class correlation coefficient of 0.988 for chew counting for a sample size of 5 participants [27]. Bites and chewing sequences were marked as food intake, whereas the remaining parts of the sensor signal were marked as non-intake. For the *k*-th annotated chewing sequence, annotated chew counts were represented by *CNT* (*k*), and the corresponding chewing rate *CR*(*k*) was computed as

(1) $$CR\;(k)=\frac{\mathrm{CNT}\;(k)}{D\;(k)},$$

where *D(k)* is the duration (in seconds) of *k-*th chewing sequence. The cumulative/total number of annotated chews for each experiment/visit and average chewing rate were represented by $A_{\mathrm{CNT}}\;(n)=\sum_{k=1}^{N}\mathrm{CNT}\;(k)$ and $A_{CR}\;(n)=\frac{1}{N}\sum_{k=1}^{N}\text{CR}\;(k)$, respectively, where *N* is the number of chewing sequences in the *n*-th experiment/visit. The resultant annotated data was used for the development of chew counting algorithms as well as for training and validation of the classification methods.

### 2.3. The Chew Counting Algorithm

**Figure 3:**
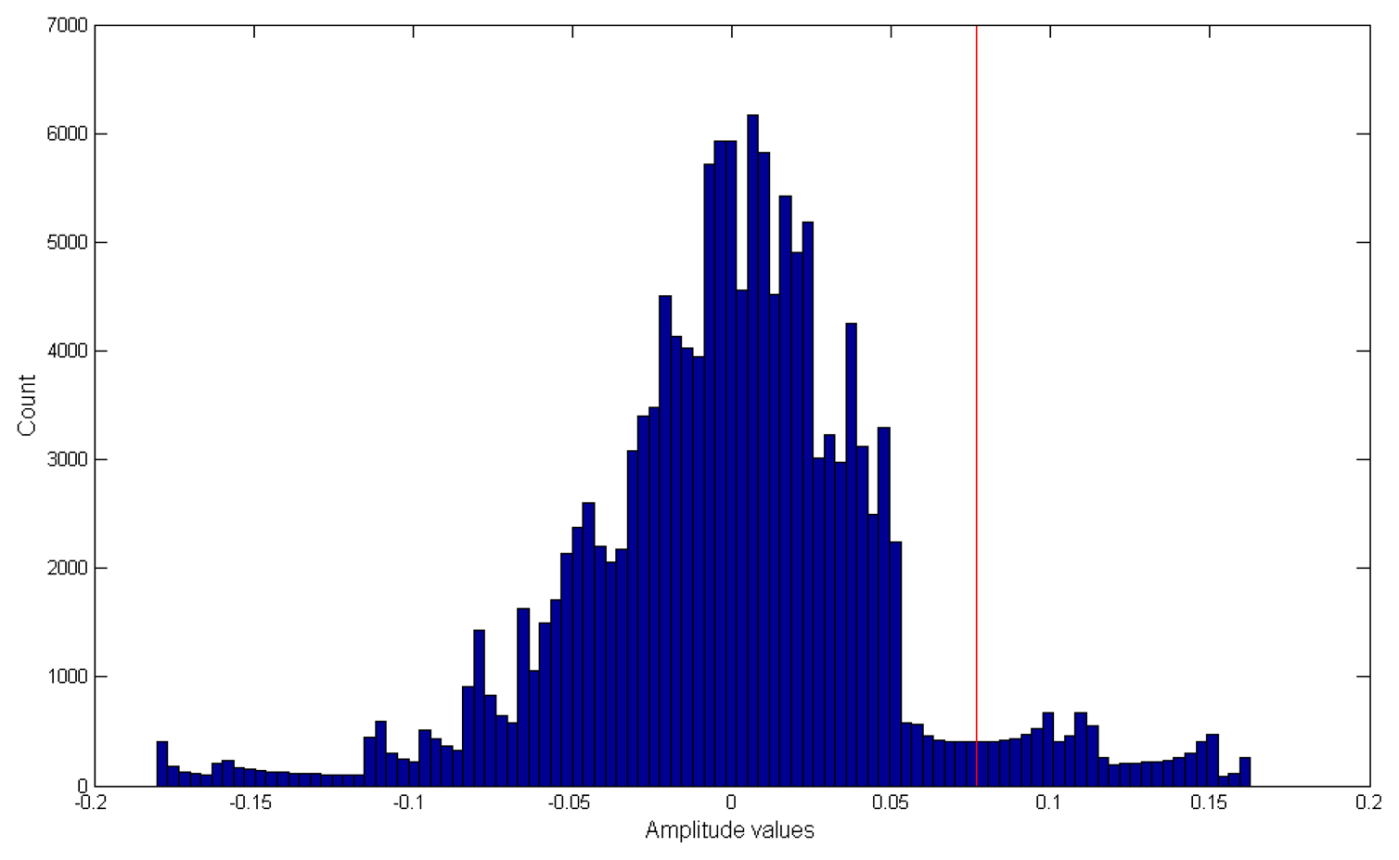
Histogram of a chewing sequence used for the selection of the threshold (*T*) for peak detection. Leave-one-out cross-validation was performed for the selection of the threshold based on the α-th percentile. The red line shows the selected threshold where only values above the threshold are considered for peak detection.

**Figure 4:**
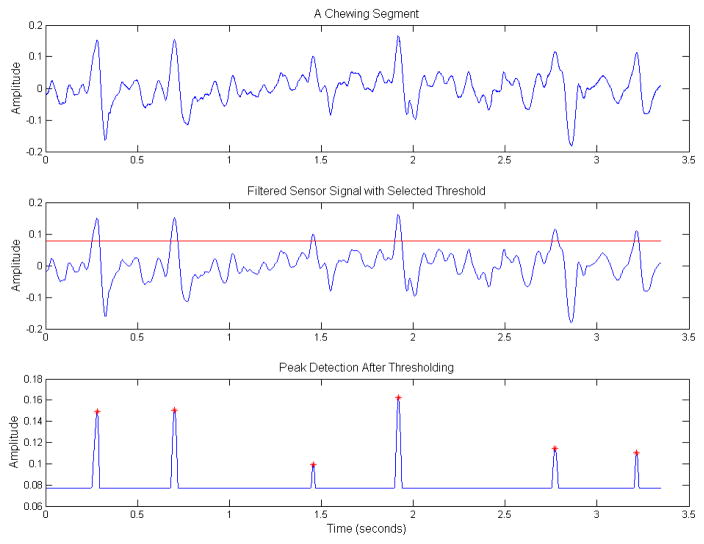
Different stages of signal processing for peak detection in chew counting algorithm. The first row shows the raw sensor signal. The second row shows filtered signal with selected threshold value (horizontal red line). The third row shows signal after thresholding and smoothing. Detected peaks are indicated by a red ‘*’.

To compute the chew counts, the sensor signals were processed in the following manner. All sensor signals were demeaned, i.e., mean amplitude were subtracted from each signal to account for offset drift in the sensor signals. Chewing frequency is in the range of 0.94 to 2 Hz; therefore, a low-pass filter with a cutoff of 3 Hz was used to remove unwanted noise [28]. Mastication/chewing of the food causes bending movements at the jaw which results in variations (peaks and valleys) in the piezoelectric sensor signal. A simple peak detection algorithm could be used to detect the presence of the peaks, and the number of peaks can be used for estimation of the number of chews. To avoid peaks caused by motion artifact such as head movement, a threshold-based peak detection approach was used where peaks were only considered if they were above a given threshold *T*. For selection of *T*, a histogram-based approach was adopted, where signal amplitudes were considered as candidates for peaks if they were in the upper α*^th^* percentile (details in Section 2.4). Figure 3 shows an example of histogram-based peak detection algorithm where the red line indicates the selected *T*-value based on the α*^th^* percentile. In order for a given amplitude value to be considered as a peak, the value needs to be higher than the selected *T*-value in the histogram (Figure 3). This example histogram was generated using a single chewing sequence (note sampling frequency is 44,100 Hz). Next, a moving average filter of 100 samples was used to smooth the resultant signal to account for small amplitude variations. The number of peaks in the resultant signal gave an estimate of the number of chews in a given segment. Figure 4 shows an example of the chew counting algorithm. For the *n^th^* experiment/visit, the cumulative estimated chew count is given by *E_CNT_(n)*. For performance evaluation of the proposed chew counting algorithm, the estimated chew counts were compared to the manually annotated chew counts, and errors were computed for each participant (all visits). Both mean errors and mean absolute errors were reported as

(2) $$\mathrm{Error}=\frac{1}{M}\sum_{n=1}^{M}\left[\frac{(A_{\mathrm{CNT}}\;(n)-E_{\mathrm{CNT}}\;(n))*100}{A_{\mathrm{CNT}}\;(n)}\right],\;\text{and}$$

(3) $$|\mathrm{Error}|\;=\frac{1}{M}\sum_{n=1}^{M}\left|\frac{(A_{\mathrm{CNT}}\;(n)-E_{\mathrm{CNT}}\;(n))*100}{A_{\mathrm{CNT}}\;(n)}\right|,$$

where *M* was the total number of experiments (visits) in this case. This algorithm was used in two different approaches, i.e., a semi-automatic approach and a fully automatic approach. In the semi-automatic approach, manually annotated chewing segments from the sensor signal were considered for chew counting. In the fully automatic approach, the automatic recognition of food intake (chewing segments) preceded the application of the chew counting algorithm.

### 2.4. Semi-Automatic Approach: Parameter Determination and Validation

In the semi-automatic approach, manually annotated chewing segments were used with the chew counting algorithm. Large amplitude variations were observed in the sensor signal due to different levels of adiposity in the study participants, variations in the sensor placements, and variations in the physical properties of food items requiring different chewing strengths. The peak detection threshold *(T)* was adapted to the signal amplitude as *T = PERCENTILE(x(k), α),* where *x(k)* is the *k^th^* manually annotated chewing sequence in the sensor signal and α ∈ [0.80, 0.97]. To avoid the over-fitting of the threshold, a subset of 20 randomly selected visits was used with leave-one-out cross-validation to find the value of α, which gave the minimum mean absolute error (Equation (3)). Data from one visit were withheld, and data from the remaining visits were used to find the value of α, which gave the least mean absolute error by performing a grid search on α in the given range. The selected value of α was used for computing the threshold *T* for the withheld visit. This process was repeated 20 times such that each visit was used for validation once, resulting in 20 different values of α. An average of these 20 different α values was calculated, and the resultant α was used for chew counting in both semi-automatic and fully automatic approaches. In the semi-automatic approach, for the *k^th^* chewing sequence in a visit, the estimated chew counts *CNT* (*k*) were used for computing corresponding chewing rate *CR* (*k*) using Equation (1). For the *n^th^* visit, for known chewing sequences, cumulative chew counts and average chewing rate were given by $E_{\mathrm{CNT}}(n)=\sum_{k=1}^{N}\mathrm{CNT}\;(k)$ and $E_{CR}(n)=\frac{1}{N}\sum_{k=1}^{N}CR\;(k)$, respectively. For the semi-automatic approach, the resultant mean error(signed) and mean absolute error (unsigned) were computed using Equations (2) and (3).

### 2.5. Fully Automatic Approach: Feature Computation and Classification

The fully automatic approach first recognizes periods of food intake by splitting the sensor signals into short segments called epochs and then applying the chew counting algorithm to the epochs labeled as food intake. The duration of the epochs was estimated to be 5 s based on the following considerations. The average length of a manually annotated chewing sequence was 7.35 ± 5.16 s; therefore, 5-s epochs were able to capture most of the chewing episodes. The frequency of chewing was in the range of 0.94 to 2.0 Hz; therefore, a 5-s epoch ensures that, even for the lower bound of chewing frequency, the epoch will contain multiple chewing events.

This approach results in epochs where some samples may belong to chewing and the rest may not belong to chewing, and vice versa. Such a situation will most likely occur at the start or end of chewing sequences. For this, a 50% determination rule was used to assign a class label for a given epoch, i.e., *C_i_* ∈ {‘−1’, ‘+1’}. An epoch was labeled as food intake (*C_i_* = +1) if at least half of the samples in the epoch belonged to food intake (based on human annotation); otherwise, it was marked as a non-intake epoch (*C_i_* = −1). For the *i^th^* epoch *x(i),* 38 time and frequency domain features were computed to find the corresponding feature vector *f_i_*. The set of features used here is the same as the feature extracted from the piezoelectric strain sensor presented in [13]. Each feature vector *f_i_* was associated with its corresponding class label *C_i_*.

ANN is a supervised machine learning technique, which has been shown to perform well in a wide range of classification problems. Advantages of ANN include robustness, flexibility, and the ability to create complex decision boundaries and handling of noisy data [13]. For classification of the epochs, a leave-one-out cross-validation scheme was used to train ANN models [13] that differentiate chewing and non-chewing. Non-chewing can be anything, e.g., absence of chewing, rest, speech, motion artifacts, etc. The classification approach using neural networks has been demonstrated to be robust in free living conditions in its ability to differentiate between chewing and non-chewing in the unrestricted environment [13] and has been shown to be superior to other methods (such as support vector machine (SVM) [12]. Three layers feed-forward architecture was used for training ANN models with a back-propagation training algorithm. The input layer had 38 neurons (for each feature), whereas the second layer (hidden layer) had 5 neurons (details described below), and the third layer (output layer) consisted of only one output neuron to indicate the predictor output class label *C_i_* (‘−1’or ‘+1’) for any given feature vector *f_i_*. Both hidden and output layers used hyperbolic tangent sigmoid for transfer function. Training and validation of the models was performed with the Neural Network Toolbox available in Matlab R2013b (The Mathworks Inc., Natick, MA, USA).

Classifier performance was evaluated in terms of *F*1 score which is defined as

(4) $$F1=\frac{2*\mathrm{Precision}*\mathrm{Recall}}{\mathrm{Precision}+\mathrm{Recall}},$$

(5) $$\mathrm{Precision}=\frac{TP}{TP+FP},\;\text{and}$$

(6) $$\mathrm{Recall}=\frac{TP}{TP+FN},$$

where *TP* is the number of true positives, *FP* is the number of false positives, and *FN* is the number of false negatives. For the leave-one-out cross-validation approach, data from one participant was used for validation (testing), whereas data from the remaining participants was used for training. This process was repeated for each participant.

An iterative approach was used to choose the number of neurons in the hidden layer. For this purpose, 30 visits were randomly selected (one visit from each participant) instead of the whole dataset to avoid over fitting. Number of neurons in the hidden layer was varied from 1 to 15 and for each neuron setting; 30-fold cross-validation was performed to compute the corresponding *F*1 score. For neural networks, the initial weights and biases are randomly assigned, which resulted in slightly different solution each time. To achieve generalizable results, the cross-validation procedure was repeated 10 times. An average of 10 iterations was computed to obtain a final *F*1-score for each fold. The final *F*1-score for each hidden neuron setting was computed by taking the average of all 30 visits. From the results, it was observed that the computed *F*1-score increases up to 5 neurons in the hidden layer, and the *F*1-score changes are small after adding more than 5 neurons. Therefore, 5 neurons were used for training final classification models.

ANN models classified epochs as “food intake” and “no-intake”. A chew counting algorithm was used for epochs classified as food intake to estimate per-epoch chew counts (*CNT* (*i*)). For the *i-th* epoch, the chewing rate (*CR* (*i*)) was computed using Equation (1). For an epoch-based approach, cumulative estimated chew counts and average chewing rate for the *n^th^* visit were computed as $E_{\mathrm{CNT}}(n)=\sum_{i=1}^{K}\mathrm{CNT}\;(i)$ and $E_{CR}(n)=\frac{1}{K}\sum_{i=1}^{K}CR\;(i)$, respectively, where *K* represents the total number of epochs in a visit. Errors (both signed and unsigned) for chew counts were computed using Equations (2) and (3). For chew counting algorithms (in both semi- and fully automatic approaches), a 95% confidence interval was used to find the interval for the mean to check if the true mean error represents underestimation or over-estimation.

To compare the total number of chews estimated by the semi-automatic and fully automatic approaches with the manually annotated chew counts, a one-way analysis of variance (ANOVA) was used. The null hypothesis in this case was that the means of chew counts estimated (for all visits) by all approaches (manually annotated, semi-automatic and fully-automatic approaches) are the same, whereas the alternate hypothesis suggested that the means were different. An ANOVA was also performed for comparing the performance of the proposed method for different meal types such as breakfast, lunch, and dinner.

## 3. Results

The collected dataset consisted of a total of 5467 chewing sequences marked by human raters with a total of 62,001 chews (average chews per meal: 660 ± 267 chews). The average chewing rate for all meals from human annotation was 1.53 ± 0.22 chews per second. Table 1 shows meal parameters such as duration, number of bites, chews, swallows, and mass ingested grouped by type of meal.

For the semi-automatic approach, the algorithm estimated total chew count of ( $\sum_{n=1}^{M}E_{\mathrm{CNT}}\;(n)$) 58,666 (average chews per meal: 624 ± 278) with an average chewing rate of 1.44 ± 0.24 chews per second. The chew counting algorithm was able to achieve a mean absolute error (Equation (3)) of 10.4% ± 7.0% for the total number of chews compared to human annotated number of chews. The average signed error was (Equation (2)) 5.7% ± 11.2%. The 95% confidence interval (CI) for the mean for the signed error was CI (3.4%, 8.0%).

**Figure 5:**
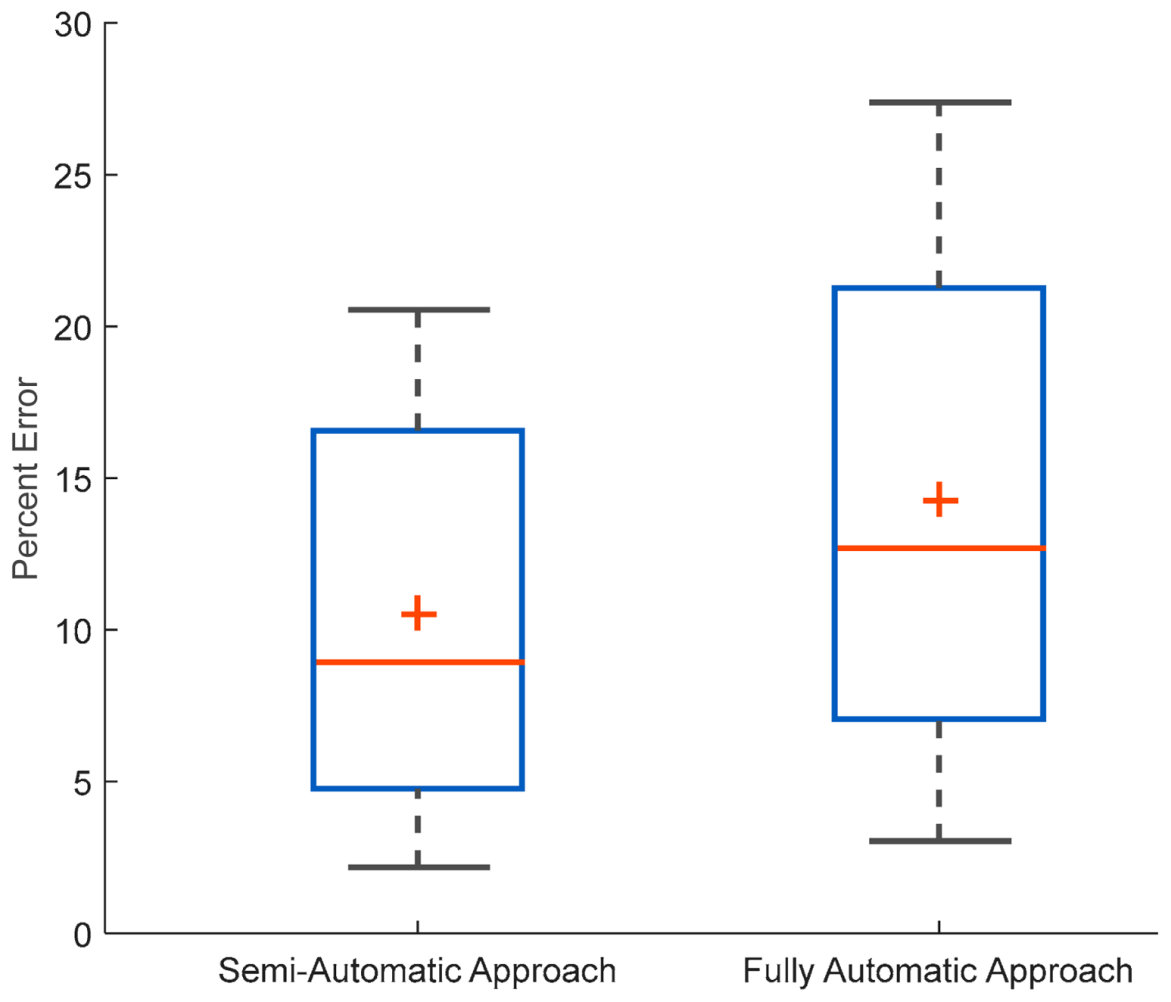
Distribution of mean absolute error of the chew counting algorithm for both semi- and automatic approaches.

**Figure 6:**
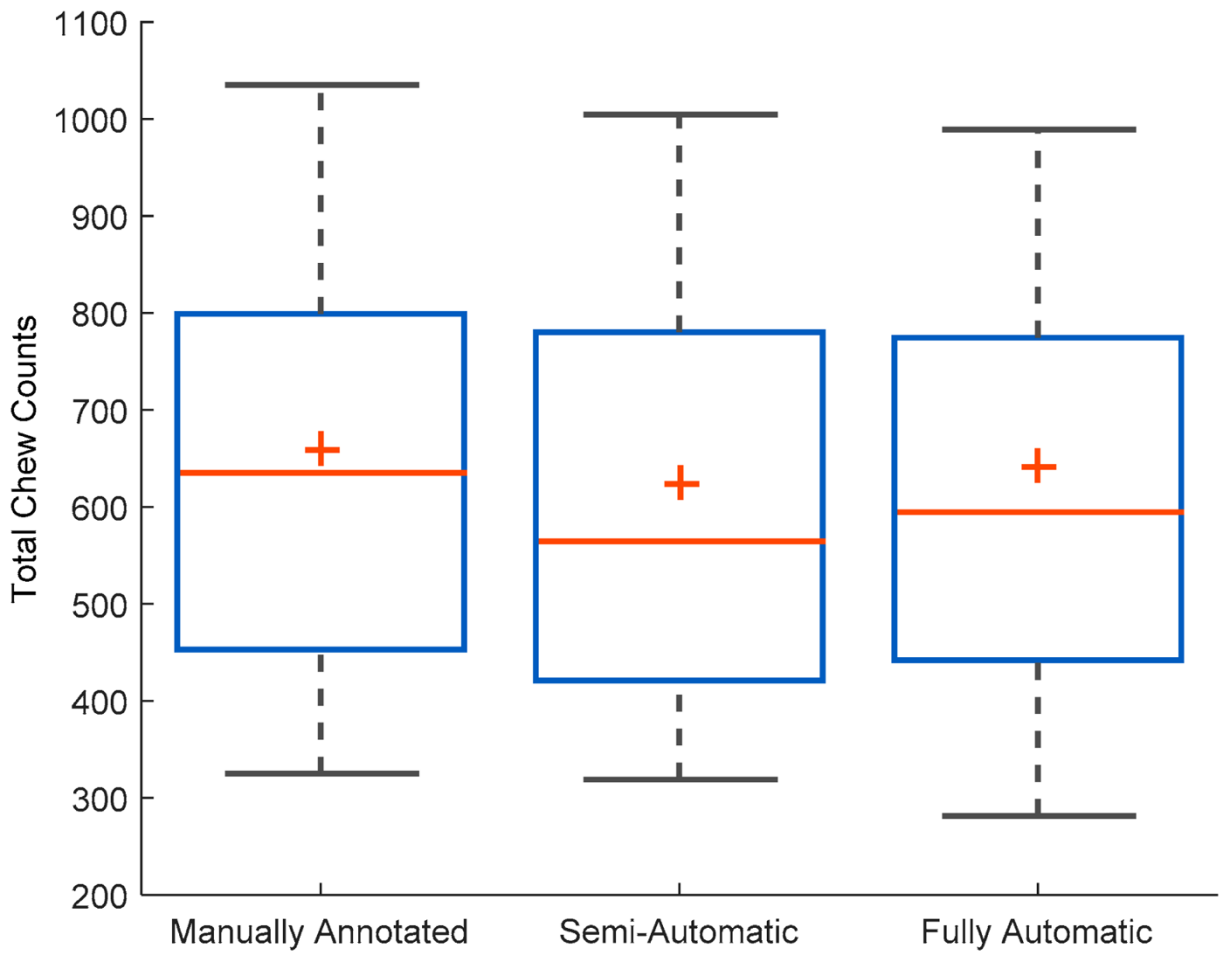
Box plots for total number per meal by human annotation; algorithm estimation with manually annotated, semi-automatic approach and fully automatic approaches.

In the fully automatic approach, trained ANN models were able to detect food intake with an average *F*1 score of 91.0% ± 7.0% with the average precision and recall of 91.8% ± 9.0% and 91.3% ± 8.8% respectively, using leave-one-out cross-validation. Further application of the chew counting algorithm resulted in 59,862 total chews, 636 ± 294 average chews per meal, and an average chewing rate of 1.76 ± 0.31 chews per second. The mean absolute error was 15.0% ± 11.0%. In this case, the average signed error was 4.2% ± 18.2%. The 95% confidence interval (CI) for the mean for the signed error was CI (0.05%, 8.10%). Figure 5 shows the distribution of both mean absolute errors for the semi- and fully automatic approaches. Figure 6 shows the distribution of chew counts per meal for human annotated chews, the estimate chew counts from the chew counting algorithm for semi- and fully automatic approaches.

Table 2 shows the results of the ANOVA for comparing the mean chew counts among manually annotated, semi-automatic and fully-automatic approaches. Results of the statistical analysis showed no significant differences between the mean chew counts among different methods (*p*-value (0.68) > 0.05). Table 3 shows average errors in the chew count estimation of both approaches for breakfast, lunch, and dinner meals. Results of ANOVA show that there were no significant differences among different meal types (the semi-automatic approach *p*-value: 0.87 > 0.05; the fully-automatic approach *p*-value: 0.28 > 0.05).

## 4. Discussion

This work presented a method for automatic recognition and quantification of chewing from piezoelectric sensor signals in terms of chewing counts and chewing rates. Results of the chew counting algorithm for both semi- and fully automated approaches suggest that the method proposed can provide objective and accurate estimation of chewing behavior. The method was tested on a comparatively large population and with a wide variety of food.

In the semi-automatic approach, the algorithm was used to estimate chew counts and chewing rates in manually annotated chewing segments and was able to achieve a mean absolute error of (Equation (3)) 10.4% ± 7.0%. For this approach, the mean signed error (Equation (2)) in comparison with human chew counts was 5.7% ± 11.2%. A 95% CI computed for signed error (3.43%, 8.02%) did not include zero with both limits being positive; therefore, the results show a trend of underestimation of the chew counts. A possible reason for this trend is the variability of food properties requiring different strength of chewing and variability in individual chewing patterns.

The fully automatic approach presents a more realistic use of the proposed method for automatic detection and quantification of chewing behavior. In the proposed method, ANN models for food intake detection preceded the use of the chew counting algorithm for epochs classified as food intake. This two-stage process resulted in a higher mean absolute error for chew counting (15.0% ± 11.0%) compared to the semi-automatic approach. The increase in error was expected as the error from both the classification stage and the chew counting stage accumulate. Other methods for chew count estimation from sensors have reported similar errors, e.g., in [9], the authors proposed the use of an accelerometer placed on the temporalis muscle and achieved a mean absolute error of 13.80 ± 8.29 for chew counts from four participants eating small pieces of watermelon. An acoustic-based chewing event counting approach using in-ear microphones presented in [8] reported an overall recall of 80% with precision at 60%–70% for three different food items. However, the approach presented in this work was tested on a larger population and with a wider variety of foods compared with the other reported results.

For cumulative chew counts, results of the one-way ANOVA (Table 2) show that the differences between the mean chew count for each experiment/visit were not statistically significant, i.e., there was not enough evidence to reject the null hypothesis (all means are equal) at the given *p*-value of 0.68. This shows that the proposed algorithm can provide chew counts that are close to the reference chew counts obtained by human raters from video observation. In this work, human annotated chew counts were used as the gold standard for algorithm development. Video-based annotation has been used by a number of researchers as the gold standard for algorithm development in food intake monitoring research [8,9,15]. The *p*-value also indicates that the trend of underestimation of the chew counts by the algorithm is not statistically significant and can be ignored based on the strong evidence (given by *p*-value). Table 3 shows that the mean absolute error was also independent of the meal type, i.e., breakfast, lunch, or dinner. This shows that the proposed method can be used for the estimation of chew counts for foods traditionally consumed for breakfast, lunch, or dinner without any sacrifice of the performance.

An average chewing rate for manually annotated chewing sequences was 1.53 chews per second, which is in the range of chewing frequencies reported in the literature (0.94–2.5 chews per second) [28]. The chewing rates estimated by the chew counting algorithm were also in the range of previously reported values. The fully automatic chew counting approach resulted in the highest chewing rate of 1.76 with the standard deviation of 0.31 chews per second. A potential reason is that the threshold for each epoch is a function of the signal amplitude in that particular epoch. Amplitude variations in epochs misclassified as food intake are smaller compared with the actual chewing; therefore, those particular epochs can result in higher chew counts. One possible improvement is to calculate global thresholds for each participant to avoid higher chew counts in epochs incorrectly classified as food intake. This will require the participant-dependent calibration of threshold computation.

In both semi- and fully automatic approaches, errors were computed for cumulative chew counts for each experiment/visit rather than the chews per known chewing segment. In the epoch-based approach, one chewing sequence may be divided into multiple epochs, and there is a possibility that a part of chewing sequence may be labeled as non-chewing epochs and vice versa (because of the boundary conditions mentioned above); therefore, a direct comparison between these two approaches was not possible. To tackle this issue, the comparison was performed for the total number of chews for each visit (meal).

One of the strengths of this study was the testing of the sensor, the signal processing, and the pattern recognition algorithms in the multi-meal experiments with a relatively larger population, where the daily eating behavior of people was replicated. Meals were kept as natural as possible. Participants were allowed to talk during the meals, and there were no restrictions on the body movements while sitting, such as head movements, coughing, etc. This enabled the inclusion of naturally occurring activities that are performed by individuals who are sitting, and it adds to the robustness of the classifications algorithm. Participants had the choice of selecting different meal times, i.e., breakfast, lunch, or dinner, and the choice of food items available at the cafeteria to make two different meals. While the food selection for a particular participant was somewhat limited, the study in general included a wide variety of food items with varying physical properties that may affect chewing. While not directly tested in this study, it is expected that the foods with different physical properties will have a different chew estimation error, which may average out across multiple food types over an extended period of time. This hypothesis will be tested in future studies.

All presented methods were developed as participant-independent group models that do not need individual calibration. Since each participant had different meal content and chewing patterns, the use of participant-dependent models (both for chew counting as well as for training classification models) may result in better accuracy and lower error, but will require calibration to each participant.

Overall, the presented method was able to detect and characterize the chewing behavior of a group of participants consisting of a wide range of adiposity, consuming a variety of food, and having a wide range of physical properties. This system needs to be further explored and tested in free living conditions. Sensor-derived chew counts can be used for creating models to estimate mass per bites [8] and energy intake in a meal [22]. One limitation of the fully automatic approach presented here is the use of a fixed size epoch, which contributes towards the increase in error. Rather than relying on the epoch-based approach, there is a need to develop algorithms that can first separate potential chewing sequences from non-chewing sequences (without using fixed window segments) and then use classification models to identifying them as food-intake or no-intake sequences.

Another limitation is that the sensor system used in this study relied on an adhesive sensor and wired connections to the data acquisition system. Since the reported study, the jaw sensor has been implemented as a Bluetooth-enabled wireless device that has been tested in free living conditions [13]. The attachment of the sensor to the skin using a medical adhesive has been shown to be simple and robust, similar to widely used adhesive bandages. In the free living study [13], the sensor did not experience any attachment issues and continuously remained on the body for approximately 24 h. Although not tested in this study, a previous study [13] demonstrated the robustness of the classifier to differentiate between chewing and other activities such as uncontrolled motion artifacts, e.g., walking, head motion, and other unscripted activities of daily living. The user burden imposed by the sensor was evaluated in [29]. Results of the study suggested that participants experienced a high level of comfort while wearing the adhesive sensor, and the presence of the sensor did not affect their eating. For long-term use (weeks and months), the sensor system may need further modifications to increase comfort and user compliance.

## 5. Conclusions

This work presented a method for the automatic detection and characterization of chewing behavior in terms of chew counts and chewing rates. A histogram-based peak detection algorithm was used to count chews in semi- and fully automatic approaches. For the semi-automatic approach, the method was able to achieve a mean absolute error of 10.4% ± 7.0%. In the fully automatic approach, sensor signals were first divided into 5-s epochs that were classified as chewing or non-chewing by an ANN. In the fully automatic approach, a classification accuracy of 91.0% and a mean absolute error of 15.0% ± 11.0% were achieved. These results suggest that the proposed method can be used to objectively characterize chewing behavior.

## Figures and Tables

**Table 1:** Details about the duration, number of bites, chews, swallow, and mass in grams for different meal types.

	Breakfast					Lunch				

	Duration s	Mass g	Bites #	Chews #	Swallows #	Duration s	Mass g	Bites #	Chews #	Swallows #
**Mean**	815.27	618.83	43.73	508.23	70.50	1011.06	675.50	41.33	748.31	78.53
**STD**	349.75	191.54	10.83	184.61	27.39	321.11	160.06	11.52	253.76	22.88
**Min**	406	299	28	204	40	485	381	23	267	44
**Max**	1964	914	69	1036	154	1839	1188	65	1352	131

**Total**	**24,458**	**18,565**	**1312**	**15,247**	**2115**	**36,398**	**24,318**	**1488**	**26,939**	**2827**

	***Dinner***					***All Meals (Total)***				

	***Duration s***	***Mass g***	***Bites #***	***Chews #***	***Swallows #***	***Duration s***	***Mass g***	***Bites #***	***Chews #***	***Swallows #***

**Mean**	1281.89	839.79	52.14	707.68	107.96	1029.24	706.35	45.32	659.59	84.73
**STD**	645.78	281.86	19.02	294.28	39.53	481.81	228.79	14.58	266.72	33.58
**Min**	460	198	25	181	55	406	198	23	181	40
**Max**	3052	1373	109	1441	209	3052	1373	109	1441	209

**Total**	**35,893**	**23,514**	**1460**	**19,815**	**3023**	**96,749**	**66,397**	**4260**	**62,001**	**7965**

**Table 2:** Results of one-way analysis of variance (ANOVA) for comparison between different chew counting approaches (manually annotated, semi-automatic and fully automatic).

Source of Variation	Sum of Squares	Degrees of Freedom	Mean Square	F	p-Value	F-Crit
**Between Groups**	60,737.45	2	30,368.73	0.39	0.68	3.03
**Within Groups**	21,857,591.03	279	78,342.62			
**Total**	21,918,328.49	281				

**Table 3:** Mean absolute errors for different meals for both semi- and fully automatic approaches.

Meal Type	Semi-Automatic	Fully Automatic
**Breakfast**	10.90% ± 7.59%	17.58% ± 12.95%
**Lunch**	10.30% ± 7.03%	12.65% ± 10.74%
**Dinner**	9.99% ± 6.63%	15.09% ± 9.19%
**Overall**	10.40% ± 7.03%	15.01% ± 11.06%

## Abbreviations

The following abbreviations are used in this manuscript

ANN: artificial neural network
*N*: experiment/visit number
*K*: manually annotated chewing sequence
*i*: epoch number
*CNT*: chew counts
*CR*: chewing rate
*A_CNT_(n)*: total annotated chew counts for n^th^ experiment/visit
*E_CNT_(n)*: total estimated chew counts for n^th^ experiment/visit
|*Error*|: mean absolute error (unsigned)
*Error*: mean error (signed)
